# Items

**Published:** 1883-11

**Authors:** 


### Items.

Mr. Edgar L. Wakeman, the well-known Chicago representative of the Louisville *Courier-Journal*, will begin the publication in this city on Christmas day of the *Current*, a weekly literary journal. The venture of Mr. Wakeman is unique, and promises to be an event in the literary world. His intimate friends who have known of his purposes for some time, and have been made more or less familiar with his preparations for the work, are confident that in the *Current* Chicago is to have a literary publication that will do the city much honor and will make the pretentious Eastern magazines look to their laurels.

The founding of this journal has been in contemplation by its projector for ten years. Active preparatory work for the same has been in progress nearly five years.

That the choice of Chicago as its place of publication is one of correctness is shown by the hearty concurrence in that view as evidenced by the noble list of writers already pledged to its future, and the munificent material encouragement already bestowed.

The *Current* will editorially discuss, with brevity and dignity, all current topics of human interest. It will treat in special papers at greater length, but always with brevity and directness, all questions worthy of earnest inquiry. These features, it is believed, will be carried out in such a manner as to win universal regard.

Following these, the *Current* will present, in each issue, an extended and valuable resume, of an editorial character, comprising comments upon current matters of real interest in Literature, Science, Art, Music, the Drama, and all the higher professions. In these will appear papers and critiques by the best writers upon these subjects in America and Europe; from the latter, by cable, as frequently as their importance may demand.

Aside from these features, there will appear in every issue of the *Current*, it is confidently believed, a greater variety and a higher grade of general literature, of a clean, noble, fascinating, and elevating character, than is now given in any periodical extant.

As an exact assurance that this is not only promised, but has already been arranged for, and will be carried out to the letter, an editorial staff, of exceptional ability and strength, has been for a considerable time under engagement. Among those already secured are the following:

Rev. Dr. Joseph Parker, Alphonse Daudet, Robert Collyer, Emile Zola, Dr. De Griez Von Ronse, Henrik Ibsen, William Henry Smith, Lucy H. Hooper, Joaquin Miller, Henry Watterson, William Young, Charles Loring Brace, Paul H. Hayne, John Ruskin: L. Alma Tadema, Hjalmer Hjorth Boyeson, Alvin P. Hovey, John Habberton, Dr. Edouard Lasker, Ignatius Donnelly, Xavier De Montepin, William F. Vilas, Bjornsterne Bjornson, George W. Cable, Edouard Remenyi, Edouard Hanslick, William Moseley Hall, Carl Fr. Mayer, Tony Revillon, Carlotta Perry, Rev. E. P. Roe, Canon Farrar, Dr. Paul Lindau, James Walter, John Burroughs, M. Georges Clemenceau, William F. Poole, Prof. R. S. Anderson, Lionel Tennyson, Lady Lindsay, Austin Dobson, Laboulaye (unpublished MSS.), etc.

